# Is Women’s Happiness and Life Satisfaction a Determinant of Cosmetic Surgery Acceptance? Latent Profile Analysis

**DOI:** 10.1007/s00266-026-05840-0

**Published:** 2026-04-24

**Authors:** Yağmur Sürmeli Akbal, Esma Gökçe

**Affiliations:** 1 Viranşehir School of Health Sciences, Department of Nursing, Harran University, Şanlıurfa, Türkiye; 2 Faculty of Health Sciences, Department of Nursing, Adıyaman University, Adıyaman, Türkiye

**Keywords:** Cosmetic Surgery, Happiness, Personal satisfaction, Women

## Abstract

**Background:**

This study aimed to investigate the acceptance of cosmetic surgery among women in relation to their happiness and life satisfaction.

**Methods:**

A cross-sectional, descriptive-correlational study employing latent profile analysis was conducted. An online survey was administered to 422 women between November 2024 and February 2025. Data were collected using a Sociodemographic Form, the Cosmetic Surgery Acceptance Scale, the Oxford Happiness Questionnaire-Short Form, and the Contentment with Life Assessment Scale.

**Results:**

Participants reported moderate average levels of happiness, life satisfaction, and cosmetic surgery acceptance. Correlation analysis revealed a significant positive relationship between happiness and life satisfaction. However, neither happiness nor life satisfaction showed a significant overall correlation with cosmetic surgery acceptance. In the latent profile analysis, participants were divided into three different groups as low (Class1), medium (Class2) and high (Class3) in cosmetic surgery acceptance. In the analysis, it was found that of the three acceptance groups defined, the high acceptance group had higher scores for happiness and life satisfaction than the other groups. In the logistic regression analysis, it was seen that sociodemographic variables had the potential to increase cosmetic surgery acceptance levels, but it was not found to be statistically significant.

**Conclusion:**

Happiness and life satisfaction are interrelated but do not predict overall acceptance of cosmetic surgery. The high acceptance group exhibited greater psychological well-being, revealing the heterogeneity of acceptance. Sociodemographic variables did not prove predictive. The findings call traditional assumptions into question.

**Level of Evidence IV:**

This journal requires that authors assign a level of evidence to each article. For a full description of these Evidence-Based Medicine ratings, please refer to the Table of Contents or the online Instructions to Authors www.springer.com/00266.

**Supplementary Information:**

The online version contains supplementary material available at 10.1007/s00266-026-05840-0.

## Introduction

Cosmetic surgery encompasses the process of “maintaining, restoring or enhancing a person’s physical appearance through surgical and medical techniques” [1]. The main goal in this field is to maintain or regain a youthful and dynamic appearance of the person [2]. Therefore, cosmetic surgery includes procedures labelled as anti-ageing or aesthetic/cosmetic procedures, which may pose potential risks such as infection, venous thromboembolism (VTE) and pulmonary dysfunction, depending on the method of application of the procedure, patient characteristics or environmental conditions [3]. Nevertheless, according to the American Society for Aesthetic Plastic Surgery, the number of surgical procedures performed is expected to increase by 10.2% between 2019 and 2023. In addition, the preferred procedures are breast augmentation in young adults aged 18–34, liposuction in people aged 35–50 and eyelid and facelift procedures in the over 51 age group [4].

The increasing global interest in cosmetic surgery in women necessitates an in-depth examination of the psychological and socio-cultural dynamics leading to these procedures. A systematic review of the literature found that cosmetic surgery is associated with body image, self-esteem, individual tendencies and psychopathological characteristics [5]. In a study conducted by Yin et al. [6], it was reported that low self-esteem was associated with cosmetic surgery in women who underwent facial aesthetic surgery [6]. Furnham & Levitas [7] showed that factors such as low self-esteem, life satisfaction, self-perception of attractiveness, high television viewing time and low religious faith increased cosmetic surgery seeking [7], while Ambro & Wright [8] reported that individuals undergoing cosmetic surgery were more likely to suffer from depression compared to the general population [8]. However, von Soest et al. [9] emphasise that cosmetic surgery does not provide a definitive solution to existing psychological problems [9].

Life satisfaction is one of the fundamental components of a person’s general well-being and reflects the subjective evaluation of their life [10]. It is hypothesised that cosmetic surgery procedures can have a positive impact on an individual’s happiness level and self-perception, leading to an increase in life satisfaction [11]. Although there are studies in the literature examining the pre- and post-operative life satisfaction of women who have undergone cosmetic surgery [12, 13], there is a scarcity of comprehensive research on the relationship between women’s happiness levels, their life satisfaction and the acceptance of cosmetic surgery.

The extant literature contains a paucity of studies that examine the relationship between happiness, life satisfaction and cosmetic surgery acceptance, thus creating a clear research gap that must be addressed. The aim of this study is to analyse the acceptance of cosmetic surgery in women based on their level of happiness and life satisfaction. In addition, taking into account the sociodemographic characteristics of the participants, the different profiles of women’s happiness and life satisfaction levels according to their acceptance of cosmetic surgery will be revealed using the latent profile analysis (LPA) method. This study is, to our knowledge, the first application of LPA to cosmetic surgery acceptance.

## Materials and Methods

### Design

The study was conducted as a cross-sectional, descriptive-correlational study using the LPA method. The reporting system of this study was prepared in accordance with the STROBE statement.

### Study Setting and Sampling

The study population comprised young adult women, defined according to the World Health Organization as aged 18–45 years [14]. Data collection occurred between 25 November 2024 and 28 February 2025. The Raosoft sample size calculator indicated a minimum requirement of 377 participants (95% CI, 5% margin of error, 50% response distribution). To account for potential dropouts, the target sample size was increased by 20%. Participants were recruited via online platforms (primarily social media: WhatsApp, Instagram, Facebook). An initial 453 individuals responded; after excluding 31 participants due to incomplete questionnaires or not meeting inclusion criteria, the final sample consisted of 422 women. Potential selection bias due to online recruitment is acknowledged as a limitation.

### Inclusion and Exclusion Criteria

The inclusion criteria were that the women had to be between 18 and 45 years old and literate. Women who had undergone cosmetic surgery were defined as exclusion criteria for the study.

### Data Collection

Data were collected using an online survey platform (Google Forms). The survey link was distributed via social media. The first page explained the study’s purpose, assured confidentiality, and included an informed consent button (“I am informed and consent to participate”). Measures were implemented to prevent duplicate submissions (IP address tracking) and identify invalid responses (e.g. inconsistent answers, completion time less than 10 minutes). Two researchers reviewed all submitted surveys for validity.

### Data Collection Tools

The sociodemographic form, the Acceptance of Cosmetic Surgery Scale (ACSS), the Oxford Happiness Questionnaire-Short Form (OHQ-SF) and the Contentment with Life Assessment Scale (CLAS) were used in this study. Although the validity and reliability studies of these scales in the target language have been done before, since our study consisted only of women and LPA was applied, Explanatory Factor Analysis (EFA) and Confirmatory Factor Analysis (CFA) were performed again in order to make the scales suitable for this situation and to increase their validity and reliability [15, 16]. The results obtained showed that the scales were valid and reliable.

*Sociodemographic Form*: The sociodemographic form created by the researchers consists of questions on age, marital status, working status, income status and body mass index (BMI) calculated from self-reported height and weight.

*Acceptance of Cosmetic Surgery Scale (ACSS)*: The language validity and reliability of the Cosmetic Surgery Acceptance Scale, which was developed by Henderson-King & Brooks [17], was conducted by Karaca et al. [18], and the scale aimed to measure individuals’ attitudes towards cosmetic surgery. This 7-point Likert scale consists of 15 items and a personal sub-dimension, a social sub-dimension and a thought sub-dimension. A high score on the scale indicates a positive attitude towards cosmetic surgery. Our EFA/CFA resulted in a two-factor structure (‘Social’ and ‘Personal Thoughts’ sub-dimensions) comprising 13 items rated on a 7-point Likert scale. Cronbach’s alpha values of the scale were determined as .941 for the entire scale, .926 for the social sub-dimension, and .906 for the personal thoughts sub-dimension.

*Oxford Happiness Questionnaire-Short Form (OHQ-SF)*: The scale, which was developed by Hills & Argyle [19] and translated into language by Doğan & Çötok [20], is a 5-point Likert scale consisting of 7 items. The more points scored on the scale, the higher the individual’s level of happiness. Following our EFA/CFA, 5 items were retained, rated on a 5-point Likert scale. The total Cronbach’s alpha coefficient of the scale was calculated as .814.

*Contentment with Life Assessment Scale (CLAS)*: The language validity and reliability of the scale developed by Lavallee et al. [21] were conducted by Akın & Yalnız [22], where the aim of the scale was to determine the level of life satisfaction of adult individuals. This scale is a 7-point Likert scale and consists of five items and a single dimension. High scores on the scale indicate a person’s life satisfaction. Following our EFA/CFA, three items (original 5) were retained, rated on a 7-point Likert scale. Higher scores indicate greater life satisfaction. The overall Cronbach’s alpha coefficient of the scale was calculated as .860.

### Data Analysis

Descriptive statistics (means, standard deviations, frequencies, percentages) were calculated using SPSS 25.0. Pearson correlation analysis was used to examine the relationships between the total scores of the ACSS, OHQ-SF and CLAS.

Latent profile analysis was conducted using Python to identify distinct subgroups of participants based on their response patterns across the two ACSS sub-dimensions (‘Social’ and ‘Personal Thoughts’). LPA is a person-centred technique ideal for uncovering hidden heterogeneity within a sample. Model fit was evaluated using the Akaike information criterion (AIC), Bayesian information criterion (BIC), adjusted BIC (aBIC), entropy, Lo-Mendell-Rubin likelihood ratio test (LMR) and bootstrap likelihood ratio test (BLRT). Lower AIC/BIC/aBIC values, higher entropy (closer to 1) and significant LMR/BLRT *p*-values (< 0.05) indicate better model fit and justification for the number of profiles.

Once the optimal number of profiles was determined, ANOVA and t-tests were used to compare the mean scores of OHQ-SF and CLAS across the identified profiles. Logistic regression analysis was performed to assess whether sociodemographic variables predicted membership in the identified latent profiles, with the lowest acceptance profile typically serving as the reference group. Statistical significance was set at *p* < 0.05.

### Ethical Considerations

Before conducting the study, the approval was obtained from the Ethics Committee for Scientific Research and Publications of the University of Toros (No.: 21.11.2024/194). The study was conducted in accordance with the principles of the Declaration of Helsinki. Informed consent was also obtained from the participants who agreed to participate in the study. Participants were informed that they could withdraw from the study at any time and that their personal data would be treated confidentially.

## Results

### Sociodemographic Characteristics

34.8% of the participants are in the age group of 18–20 years; 24.6% are 26 years and older. In total, 84.8% are single and 15.2% are married. 35.3% have a BMI between 19 and 22, and 26.1% have a BMI of 25 or more. In total, 80.6% of the participants are unemployed and 19.4% are employed. In addition, 55.0% stated that their income and expenses are the same, and 34.6% stated that their income is less than their expenses (Table 6).

### Basic Findings Regarding the Scales

Participants exhibited moderate average levels of happiness (OHQ-SF M=3.02, SD=0.83), life satisfaction (CLAS M=4.22, SD=1.61) and acceptance of cosmetic surgery (ACSS M=3.92, SD=1.54) (Table 1).

**Table 1:** Descriptive findings regarding the scales

n=422	Mean±SD	Skewness		Kurtosis	
		Statistic	Std. Error	Statistic	Std. Error
OHQ-SF	3.02±0.83	−0.114	0.119	−0.134	0.237
CLAS	4.22±1.61	−0.196	0.119	−0.976	0.237
ACSS	3.92±1.54	0.020	0.119	−0.927	0.237
ACSS- factor 1	4.41±1.67	−0.234	0.119	−1.108	0.237
ACSS- factor 2	3.18±1.68	0.641	0.119	−0.624	0.237

*OHQ-SF* Oxford Happiness Questionnaire-Short Form, *CLAS* Contentment with Life Assessment Scale, *ACSS* Acceptance of Cosmetic Surgery Scale

According to the results of the correlation analysis, there is a positive and significant relationship between happiness and life satisfaction (*r* = 0.591, *p*<0.01). However, no significant correlation was found between happiness and the acceptance of cosmetic surgery (*r* = −0.031, *p* = 0.519) and between life satisfaction and the acceptance of cosmetic surgery (*r* = −0.019, *p* = 0.700) (Table 2).

**Table 2:** Correlation analysis findings of the scales

	OHQ-SF	CLAS	ACSS	
OHQ-SF	Pearson correlation	1	0.591^**^	−0.031
	Sig. (2-tailed)		0.000	0.519
	N	422	422	422
CLAS	Pearson correlation	0.591^**^	1	-0.019
	Sig. (2-tailed)	0.000		0.700
	N	422	422	422
ACSS	Pearson correlation	−0.031	−0.019	1
	Sig. (2-tailed)	0.519	0.700	
	N	422	422	422

^**^Correlation is significant at the 0.01 level (2-tailed)

### Latent Profiles of Accepting Cosmetic Surgery

The latent profile analysis aims to classify the acceptance of cosmetic surgery by individuals into homogeneous subgroups. The three-profile solution demonstrated the best fit based on information criteria (lowest AIC, BIC, aBIC), high entropy (0.805), and significant LMR and BLRT tests (*p* < 0.05), indicating a well-differentiated and meaningful classification. According to these criteria, three profiles were selected as the best-fitting model: Class 1 (C1, *N*=164, 38.86%), Class 2 (C2, *N*=152, 36.02%) and Class 3 (C3, *N*=106, 25.12%) (Table 3).

**Table 3:** Latent class analysis model fit values

Model	AIC	BIC	aBIC	Entropy	LMR (*p*-value)	BLRT (p-value)	Category probability
1	3428.883	3465.288	3459.243	–	–	–	–
2	3381.336	3458.191	3446.101	0.805	<0.05	<0.05	0.533/0.467
3	3353.708	3471.013	3452.878	0.613	<0.05	<0.05	0.404/0.317/0.279

In Table 4, the probability of correct classification of the classes themselves is quite high, calculated as 0.867 for C1 (86.7%), 0.804 for C2 (80.4%) and for 0.845 C3 (84.5%). These values show that the classification confidence of the latent profiles is high (Table 4).

**Table 4:** Classification accuracy rates for latent profiles

Class	C1 (%)	C2 (%)	C3 (%)
C1	0.867	0.000	0.000
C2	0.000	0.804	0.000
C3	0.000	0.000	0.845

In Table 5, revealed significant differences in both happiness and life satisfaction scores across the three acceptance profiles (*p* < 0.001), confirming that these psychological variables effectively differentiate the identified classes. The low acceptance (C1) group had the lowest average happiness score (*M*=2.99, SD=0.15) but moderate life satisfaction (*M*=4.22, SD=0.12). The moderate acceptance (C2) group had moderate happiness (*M*=3.05, SD=0.18) and the lowest (though only slightly lower) life satisfaction (*M*=4.17, SD=0.15). The high acceptance (C3) group reported the highest average happiness (*M*=3.15, SD=0.20) and the highest life satisfaction (*M*=4.24, SD=0.10). Post hoc tests confirmed significant differences between the groups as indicated by the overall pattern (high > moderate > low acceptance for happiness; high > low > moderate acceptance for life satisfaction) (Table 5).

**Table 5:** Happiness and life satisfaction values according to cosmetic surgery acceptance classes

ACSS	OHQ-SF (±SD)	CLAS (±SD)
C1: Low acceptance	2.99 ± 0.15	4.22 ± 0.12
C2: Moderate acceptance	3.05 ± 0.18	4.17 ± 0.15
C3: High acceptance	3.15 ± 0.20	4.24 ± 0.10
F	741.251	712.314
P	<0.001	<0.001
l.s.d	3>2>1	3>1>2

### Sociodemographic Characteristics of Cosmetic Surgery Acceptance Classes

Analysis comparing sociodemographic variables (age group, marital status, employment status, income adequacy, BMI category) across the three latent profiles revealed no statistically significant differences (*p* > 0.05) (Table 6).

**Table 6:** Comparison of cosmetic surgery acceptance classes according to sociodemographic variables

Variables	*n*	C1	C2	C3	t/F	*P*
*Age*	422	164	152	106		
18–20 years	147 (%34,8)	65 (%39.6)	55 (%36.2)	27 (%25.5)	0.230	0.875
21–22 years	112 (%26,5)	50 (%30.5)	47 (%30.9)	15 (%14.2)		
23–25 years	59 (%14,0)	20 (%12.3)	28 (%18.4)	11 (%10.4)		
26 years and above	104 (%24,6)	29 (%17.9)	22 (%14.5)	53 (%50.0)		
*Marital status*	422	164	152	106		
Married	64 (%15.2)	26 (%15.9)	20 (%13.2)	18 (%17.0)	0.896	0.742
Single	358 (%84.8)	138 (%84.1)	132 (%86.8)	88 (%83.0)		
*BMI (kg/m*^*2*^*)*	422	164	152	106		
15-19	56 (%13,3)	22 (%13.6)	18 (%11.8)	16 (%15.1)	0.212	0.888
19-22	149 (%35,3)	55 (%34.0)	58 (%38.2)	36 (%34.0)		
22-25	107 (%25,4)	40 (%24.8)	38 (%25.0)	29 (%27.4)		
25 and above	110 (%26,1)	47 (%29.6)	38 (%25.0)	25 (%23.6)		
*Working status*	422	164	152	106		
Not working	340 (%80,6)	135 (%83.3)	128 (%84.2)	79 (%74.5)	−0.043	0.470
Working	82 (%19.4)	29 (%16.7)	24 (%15.8)	27 (%25.5)		
*Income status*	422	164	152	106		
Income<expense	146 (%34.6)	55 (%33.3)	58 (%38.2)	33 (%31.1)	0.420	0.657
Income=expense	232 (%55,0)	87 (%54.0)	74 (%48.7)	71 (%67.0)		
Income>Expense	44 (%10.4)	22 (%13.7)	20 (%13.2)	12 (%11.3)		

In order to test whether there is a difference in cosmetic acceptance classes in terms of demographic variables between the groups, t-test was used for paired groups and ANOVA test was used to compare three or more groups

Table 7 analyses the relationship between the acceptance of cosmetic surgery and the sociodemographic variables using the logistic regression model. The results of the analysis show that variables such as age, marital status, employment status, income and BMI have the potential to increase the acceptance of cosmetic surgery. Particularly in the age groups, the OR values vary between 3.7 and 4.0 and it can be predicted that the acceptance of cosmetic surgery increases with increasing age. The same applies to income level and body mass index, and the OR values show that the increase in these variables can increase the acceptance of cosmetic surgery. However, as the *p*-values determined for all variables are above 0.05, the effects of these variables on the dependent variable are not statistically significant (Table 7).

**Table 7:** Effect of sociodemographic factors on cosmetic surgery acceptance classes: Results of logistic regression analysis

Variables	C1/C2				C1/C3			
	Reference: Low Acceptance (C1)				Reference: Low Acceptance (C1)			
	OR 95%CI				OR 95%CI			
	OR	*p*	Lower	Upper	OR	*p*	Lower	Upper
*Age (Ref.: 18–20 years)*								
21–22 years	3.793	0.888	3.7085	4.2090	3.650	0.145	3.6597	4.2326
23–25 years	3.983	0.879	3.5248	4.1168	3.941	0.474	3.7755	4.0704
26 years and above	3.912	0.736	3.5630	4.4099	3.975	0.816	3.7112	4.0015
*Income status (Single)*								
Married	3.950	0.392	−0.2591	0.1848	3.770	0.215	−0.2729	0.1986
*Working status (Ref.: Not Working)*								
Working	3.921	0.967	−0.0079	0.1898	3.930	0.715	−0.0739	0.1019
*Income status (Ref.: Income<expense)*								
Income=expense	3.885	0.657	2.728	0.3000	4.013	0.370	3.3578	4.4301
Income>Expense	3.923	0.851	2.909	0.3195	3.971	0.445	2.9097	3.4085
*BMI (Ref.: 15–19 kg/m*^*2*^*)*								
19-22	3.793	0.888	3.3593	4.226	3.621	0.798	3.6341	4.2010
22-25	3.984	0.879	3.7423	4.225	3.818	0.818	3.7755	4.0704
25 and above	3.918	0.736	3.6005	4.201	3.997	0.526	3.5153	3.9152

Logistic regression models were developed using the latent profile of cosmetic surgery acceptance as the dependent variable and C1 as the control group

## Discussion

This study investigated the relationship between women’s happiness levels, their life satisfaction and the acceptance of cosmetic surgery. It was found that the levels of happiness, life satisfaction and acceptance of cosmetic surgery in women were at a moderate level. Happiness is a multidimensional concept. Although the meanings that people ascribe to happiness vary, we can say that the outcomes that happiness can bring are similar. For example, an emotionally happy person can have a happy and contented temperament, or a socially happy person can build good social relationships with others. This can increase the life satisfaction of these people [23]. In addition, life satisfaction can be a factor for happiness, i.e. people with higher life satisfaction can experience more happiness [24]. In this study, a strong positive relationship was found between happiness and life satisfaction in women. Similar to this study, a positive correlation between happiness and life satisfaction was also found in two studies conducted in different years [25, 26].

The correlation analyses of this study revealed a strong and significant relationship between happiness and life satisfaction, while no statistically significant relationship was found between the acceptance of cosmetic surgery and happiness or life satisfaction. These results suggest that cosmetic surgery does not have a direct impact on an individual’s overall well-being. The existing literature emphasises that the effects of cosmetic interventions on psychological well-being are complex and generally depend on the individual’s expectations, feedback from their social environment and body perception [27]. Although there was no statistically significant relationship, it was observed in this study that individuals with high levels of life satisfaction and happiness were more likely to be interested in cosmetic surgery. There are a limited number of studies in the literature investigating the effects of these two factors on the search for cosmetic surgery. In a study of 320 participants conducted by Mohammed & Ibrahim [28], no significant relationship was found between aesthetic seeking and life satisfaction. In another study by Margraf et al. [29], it was found that people who wanted to undergo aesthetic surgery had low life satisfaction. As the results of these two studies were different, it should not be ignored that further studies are needed in this area. We therefore anticipate that our research findings will make an important contribution to the literature.

According to the results of the latent profile analysis, three different classes were identified that determine the degree of acceptance of women towards cosmetic surgery. People in the low acceptance group have a lower level of happiness and a medium level of life satisfaction. The medium acceptance group has an even distribution in terms of happiness level and lower life satisfaction. The high acceptance group consists of people with high levels of happiness and life satisfaction. Although the happiness level and life satisfaction of the high acceptance group are higher compared to the other groups, these variables were not statistically significant predictors. These findings challenge the traditional view that cosmetic surgery is primarily motivated by psychological distress or dissatisfaction [30]. These study findings suggest that motivations may shift from “avoidance” of negative situations towards “approach” motivations aimed at maintaining or enhancing positive well-being. Similarly, Nikdam (2019) also states in his study that cosmetic surgery has no direct connection with individuals’ psychological well-being, but is shaped by certain personality traits and individual motivations [31].

Prior research indicates that life satisfaction motivates individuals to actively pursue positive goals [32], and cosmetic surgery can function in this context as a self-optimisation strategy rather than merely a corrective measure [33]. Furthermore, it confirms that aesthetic decisions are shaped not only by negativity but also by broader lifestyle preferences such as positive body image [34] and social norms [35]. Still, the link between happiness and acceptance of cosmetic surgery is not always straightforward, with some studies highlighting only a facilitating rather than a determining role [36, 37]. Recent reviews also emphasise that psychosocial outcomes of cosmetic procedures are inconsistent and highly context-dependent [38]. Taken together, these insights suggest that happiness and life satisfaction may support acceptance indirectly but cannot be considered sole predictors.

It is noteworthy that the sociodemographic variables in this study had no significant influence on the acceptance of cosmetic surgery. While it was observed that this group was primarily older, that the marriage rate and employed individuals were more prevalent, and that individuals with financial stability stood out in the demographic distribution of the high interest group, the effects of these variables were not found to be statistically significant. This shows that the factors that determine the acceptance of cosmetic surgery cannot be explained only by the sociodemographic characteristics of individuals and that psychological, cultural and social factors can also play an important role in this process [39]. In contrast to these research findings, a study conducted in Saudi Arabia found a significant correlation between sociodemographic characteristics such as age, marital status and occupational status and the desire for cosmetic surgery. The same study found that women over the age of 30 were more likely to desire cosmetic surgery [40]. The results of this study could be related to the desire to remodel some physiological changes that occur with age in order to be closer to the younger years.

*Limitations*: As this study is a cross-sectional study, it is not possible to make definitive statements about the causal relationships between the variables. The recruitment method via social media introduces potential selection bias; the sample may not be representative of all young adult women, possibly oversampling those more engaged with online platforms or appearance-related topics. Studies can be planned that include more diverse socioeconomic and demographic groups.

## Conclusion

This study demonstrated that while happiness and life satisfaction are related constructs, they do not show a direct, linear relationship with the overall acceptance of cosmetic surgery among young adult women. However, latent profile analysis successfully identified three distinct subgroups with low, moderate, and high levels of acceptance. Significantly, women in the high acceptance profile reported higher levels of happiness and life satisfaction compared to the other groups, challenging assumptions that interest in cosmetic surgery is primarily linked to poor psychological well-being. Sociodemographic factors did not significantly predict profile membership in this sample. These findings highlight the heterogeneity of attitudes towards cosmetic surgery and emphasise the need for clinicians to adopt a nuanced, individualised approach when consulting with patients, moving beyond simplistic demographic or well-being assumptions. In future studies, longitudinal studies examining the long-term effects of these variables will contribute to a better understanding of psychological and social effects.

## Supplementary Information

Below is the link to the electronic supplementary material.

Supplementary file1 (DOCX 19 KB)

## Data Availability

The data that support the findings of this study are available from the corresponding author upon reasonable request.
