# Supplementary material for: Is Women’s Happiness and Life Satisfaction a Determinant of Cosmetic Surgery Acceptance? Latent Profile Analysis
Source: Aesthetic Plast Surg. 2026 Apr 24;50(13):5704–11. doi: 10.1007/s00266-026-05840-0 (PMC13433374; doi:10.1007/s00266-026-05840-0)
Supplement: Supplementary file 1 [file 266_2026_5840_MOESM1_ESM.docx]

**Acceptance of Cosmetic Surgery Scale (ACSS)**

|  | **Never**  **I disagree** |  |  |  |  |  | **I totally agree** |
| --- | --- | --- | --- | --- | --- | --- | --- |
| 1. It makes sense to have minor cosmetic surgery rather than spending years feeling bad about the way you look. | 1 | 2 | 3 | 4 | 5 | 6 | 7 |
| 2. Cosmetic surgery is a good thing because it can help people feel better about themselves. | 1 | 2 | 3 | 4 | 5 | 6 | 7 |
| 3. In the future, I could end up having some type of cosmetic surgery. | 1 | 2 | 3 | 4 | 5 | 6 | 7 |
| 4. People who are very unhappy with their physical appearance should consider cosmetic surgery as one option. | 1 | 2 | 3 | 4 | 5 | 6 | 7 |
| 5. If cosmetic surgery can make someone happier with the way they look, then they should try it. | 1 | 2 | 3 | 4 | 5 | 6 | 7 |
| 6. If I could have a surgical procedure done for free I would consider trying cosmetic surgery. | 1 | 2 | 3 | 4 | 5 | 6 | 7 |
| 7. If I knew there would be no negative side effect or pain, I would like to try cosmetic surgery. | 1 | 2 | 3 | 4 | 5 | 6 | 7 |
| 8. I have sometimes thought about having cosmetic surgery. | 1 | 2 | 3 | 4 | 5 | 6 | 7 |
| 9. I would seriously consider having cosmetic surgery if my partner thought it was a good idea. | 1 | 2 | 3 | 4 | 5 | 6 | 7 |
| 10. I would never have any kind of plastic surgery (R). | 1 | 2 | 3 | 4 | 5 | 6 | 7 |
| 11. I would think about having cosmetic surgery to keep looking young. | 1 | 2 | 3 | 4 | 5 | 6 | 7 |
| 12. If it would benefit my career, I would think about having plastic surgery.. | 1 | 2 | 3 | 4 | 5 | 6 | 7 |
| 13. I would seriously consider having cosmetic surgery if I thought my partner would find me more attractive. | 1 | 2 | 3 | 4 | 5 | 6 | 7 |
| 14. Cosmetic surgery can be a big benefit to people’s self-image. | 1 | 2 | 3 | 4 | 5 | 6 | 7 |
| 15. If a simple cosmetic surgery procedure would make me more attractive to others, I would think about trying it. | 1 | 2 | 3 | 4 | 5 | 6 | 7 |
